# A transgenic zebrafish for *in vivo* visualization of cilia

**DOI:** 10.1098/rsob.220104

**Published:** 2022-08-10

**Authors:** Hongyu Zhang, Zhuoya Huang, Liuliu LV, Yuye Xin, Qian Wang, Feng Li, Lina Dong, Changxin Wu, Philip W. Ingham, Zhonghua Zhao

**Affiliations:** ^1^ Institute of Biomedical Sciences, 1331 Local Bio-Resources and Health Industry Collaborative Innovation Center of Shanxi Province, Key Laboratory of Biomedical Shanxi Province, Shanxi University, Taiyuan 030006, People's Republic of China; ^2^ School of Life Science, Shanxi University, Taiyuan 030006, People's Republic of China; ^3^ Chemical Biology and Molecular Engineering Key Laboratory of Ministry of Education, Institute of Biotechnology, Shanxi University, Taiyuan 030006, People's Republic of China; ^4^ Department of Molecular Biology, Shanxi Cancer Hospital, Affiliated Cancer Hospital of Shanxi Medical University, Taiyuan 030013, People's Republic of China; ^5^ Central Laboratory, Shanxi Provincial People's Hospital, Affiliate of Shanxi Medical University, Taiyuan 030012, People's Republic of China; ^6^ LKC Medicine School, Nanyang Technological University, 639798, Singapore

**Keywords:** transgenic zebrafish, cilia, Nphp3, live imaging, ciliary signal peptides

## Abstract

Cilia are organelles for cellular signalling and motility. Mutations affecting ciliary function are also associated with cilia-related disorders (ciliopathies). The identification of cilia markers is critical for studying their function at the cellular level. Due to the lack of a conserved, short ciliary localization motif, the full-length ARL13b or 5HT_6_ proteins are normally used for cilia labelling. Overexpression of these genes, however, can affect the function of cilia, leading to artefacts in cilia studies. Here, we show that Nephrocystin-3 (Nphp3) is highly conserved among vertebrates and demonstrate that the N-terminal truncated peptide of zebrafish Nphp3 can be used as a gratuitous cilia-specific marker. To visualize the dynamics of cilia *in vivo*, we generated a stable transgenic zebrafish Tg (*β-actin*: *nphp3N-mCherry*)^sx1001^. The cilia in multiple cell types are efficiently labelled by the encoded fusion protein from embryonic stages to adulthood, without any developmental and physiological defects. We show that the line allows live imaging of ciliary dynamics and trafficking of cilia proteins, such as Kif7 and Smo, key regulators of the Hedgehog signalling pathway. Thus, we have generated an effective new tool for *in vivo* cilia studies that will help shed further light on the roles of these important organelles.

## Graphical abstract

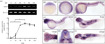

## 1. Introduction

Cilia are microtubule-based organelles found on the surface of most eukaryotic cells, where they play roles in cell motility, sensory reception and signal transduction. Cilia are classified as motile cilia or non-motile primary cilia (PC), properties reflected in their different microtubule organization [1]. Motile cilia, normally with a ‘9 + 2' microtubule structure, are found mainly on the surface of migratory cells and some functionally specialized epithelial cells where their synchronized beating facilitates cell movement or fluid clearance, respectively [2]. The PC, by contrast, is a single antenna-like organelle composed of a ‘9 + 0' microtubule structure and cell membranes attached [3]. PC are important for processes such as sensing mechanical and chemical signals from the extracellular microenvironment. They serve as a hub for the reception and processing of extracellular signals, regulating their integration with receptors and subsequent transduction of the signal inside the cell. Various molecules such as receptors, scaffolds, ion channels, adapter molecules and effector enzymes have been shown to be trafficked in PC, referred to as intraflagellar trafficking, making this organelle a vital subcellular compartment for cell proliferation, differentiation, metabolism, etc. [4–7]. Several signalling pathways in vertebrates that are critical for cell differentiation and embryonic development, including Hedgehog (Hh) and PDGF, are PC-dependent [8–11]. In humans, mutations in genes required for cilia structure or function led to cilia-associated disorders (ciliopathies), such as ciliary dyskinesia, polycystic kidney disease and craniofacial abnormalities [12–14].

Although the importance of cilia function has long been recognized, progress in their study, especially PC, has been limited by the lack of efficient methods for analysing these tiny subcellular compartments. One important goal is to elucidate the changes in cilia morphology and trafficking of cilia-localized components following the manipulation of genes or cells. To date, this has relied largely on immunohistochemistry (IHC) with fixed materials. Although much information on cilia and cilia-associated cell behaviors has been obtained in this way, the method has its limitations. The real-time dynamic changes inside of cilia *in vivo* cannot be resolved by IHC. And the approach is time-consuming and labour-intensive when troubleshooting artefacts. Implementing *in vivo* cilia labelling can in principle address these limitations and enable real-time cilia monitoring. To achieve this, a suitable cilia localization signal is needed to generate specimens in which the cilia are reproducibly labelled. While numerous proteins are localized to cilia, no conserved cilia localization signal has so far been identified. In tissue culture studies, the commonly used proteins for cilia labelling are ARL13b and serotonin receptor 6 (5HT_6_). ARL13b, a member of the ADP-ribosylation factor-like family, is a GTPase protein that anchors the ciliary membrane and plays an important role in controlling ciliary trafficking [15–19], whereas 5HT_6_ is a member of the G-protein-coupled receptor (GPCR) family and functions in regulating cilia length, dendritic morphology and cilia protein localization [20–23]. In the absence of short ciliary targeting sequences, C terminal fusions of their full-length forms to fluorescent proteins have been used for cilia labelling. However, as both proteins are essential for cilia structure and function, overexpression of either has effects on cilia, which can affect the processes being analysed [21,24–26]. In addition, the use of the transient expression for labelling purposes leads to inconsistencies among cells with different expression levels. Identification of shorter, more specific, and functionally neutral ciliary peptides could obviate these limitations.

Nephrocystin-3 (NPHP3), identified as a protein associated with nephronophthisis, a genetic ciliopathy, was reported to be localized at the Inv compartment, a distinct proximal segment of the ciliary body [27,28]. Current knowledge of Nphp3 protein shows that it is involved in biogenesis, assembly and disassembly of cilia, as well as transport of certain molecules to cilia, such as myristoylated proteins. Domain analysis indicates that the N terminal region (1–204 amino acid residues [aas]) of mouse Nphp3 is sufficient to drive the localization of the fluorescent protein to the PC in tissue culture without affecting the structure and function of cilia, indicating that it is a good candidate for a ciliary label [29]. Here, we have identified the zebrafish homolog of *nphp3* and analysed its spatio-temporal expression during embryogenesis. After confirming its N-terminal ciliary targeting sequence (CTS) peptide, a stable transgenic zebrafish line, named *sx1001*, has been established by genomic integration of *znphp3N-mCherry* driven by the *β-actin* promoter. The cilia of the *sx1001* line are uniformly labelled by red fluorescence in both embryonic stages and adult tissues, enabling live *in vivo* imaging of cilia. We present the characterization of this line and illustrate its use in analysing the processing and mechanism of the cilium during development.

## 2. Results

### 2.1. Expression analysis of zebrafish Nphp3

**Figure 1.**
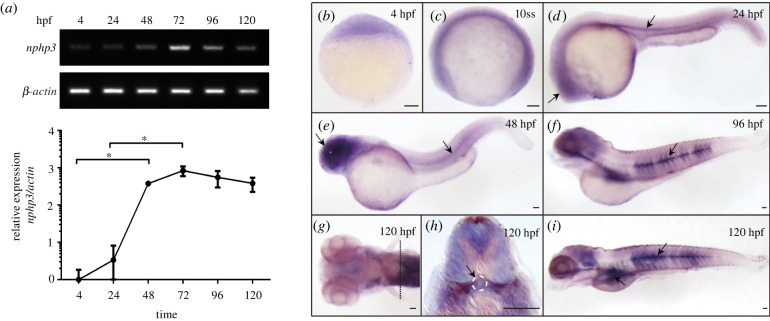
Expression analysis of zebrafish Nphp3. (*a*) The upper panel is the RT-PCR image of *znphp3* expression from 4 hpf to 120 hpf. *β-actin* was used as an internal control. The qRT-PCR was used to quantify *nphp3* mRNA levels in wild larvae as shown in the lower panel. The expression of *znphp3* was normalized by *β-actin* mRNA. The vertical coordinate is the logarithm with log10 as the base. Vertical bars represent the standard deviation (*n* = 3). One-way ANOVA was used for analysis. Significant values are noted as *p ≤ 0.05. (*b*–*i*) Whole-mount *in situ* hybridization of zebrafish *nphp3* (*znphp3*) in wild zebrafish embryos at different stages. (*b*) ubiquitous expression of *znphp3* was observed at sphere stage (4 hpf; embryo shown with animal pole to the top); (*c*) *znphp3* mRNA was uniformly distributed throughout the embryo at 10ss; (*d,e*) *znphp3* mRNA accumulated primarily in the ventral portion of the larvae, the retina, and the horizontal myoseptum region during 24–48 hpf; (*f*) the expression at the horizontal myoseptum remained and spread to the intermediated muscle fibres at 96 hpf; (*g*–*i*) *znphp3* mRNA was found at the majority of the somatic borders and was slightly expressed at the pronephros glomerulus at 120 hpf (as shown in the dotted line in *g*). Arrows point to tissues with enriched expression of *znphp3*. Scale bar, 100 µm.

We analysed the temporal expression of zebrafish *nphp3* (*znphp3*), by RT-PCR and quantitative RT-PCR of embryos and larvae from 4 hpf (hours post-fertilization) to 120 hpf. Weak maternally derived *znphp3* transcript was detected at 4 hpf, prior to the onset of zygotic transcription. An abundance of *nphp3* mRNA increased dramatically from 24 hpf to 72 hpf, then declined slightly, until 120 hpf (figure 1*a*). The spatial distribution of mRNA was analysed by *in situ* hybridization (ISH) of embryos and larvae from the 4 hpf to 120 hpf, using a DIG-labelled RNA probe against the 3′ end of the *nphp3* coding region (electronic supplementary material, table S2). Transcripts were detectable in all the stages assayed. Moderate signals were observed at sphere stage (figure 1*b*). At 10 somite stage (ss), the *znphp3* mRNA was evenly distributed throughout the embryo, while at 24 hpf, it accumulated ventrally in the retina and the horizontal myoseptum (figure 1*c,d*). This spatially regulated pattern became much more obvious at 48 hpf (figure 1*e*). The ISH signal in the retina reduced from 96 hpf to 120 hpf, while the signal at the horizontal myoseptum remained and expanded into the intervening muscle fibres. The transcript was also detected at most of the somite boundaries (figure 1*f–i*). In addition, cross sections at 120 hpf revealed weak *znphp3* mRNA could be detected in the glomerulus of the pronephros, suggesting its conserved function in kidney development (figure 1*h*).

### 2.2. Transient expression of zebrafish full-length Nphp3 and its N-segment truncation protein localized to primary cilia

Protein sequence analysis revealed that zNphp3 shares 67.04% similarity with mouse Nphp3 and 69.02% with human NPHP3 [27,29] (electronic supplementary material, figure S1A). To test whether the protein also localizes to PC in zebrafish, mRNA encoding zNphp3 fused to eGFP at its C terminus was injected into one-cell stage embryos. The zNphp3-eGFP fusion protein was robustly expressed in injected embryos and its subcellular localization was assayed by immunofluorescence (IF) staining in 18 hpf embryos. To visualize the PC, injected embryos were incubated with a fluorescently labelled antibody against acetylated tubulin (Ac-tub). Like its mouse orthologue, the tagged zNphp3 specifically localized to PC in the otic vesicle, neural tube, and myotome (electronic supplementary material, figure S1B–D).

**Figure 2.**
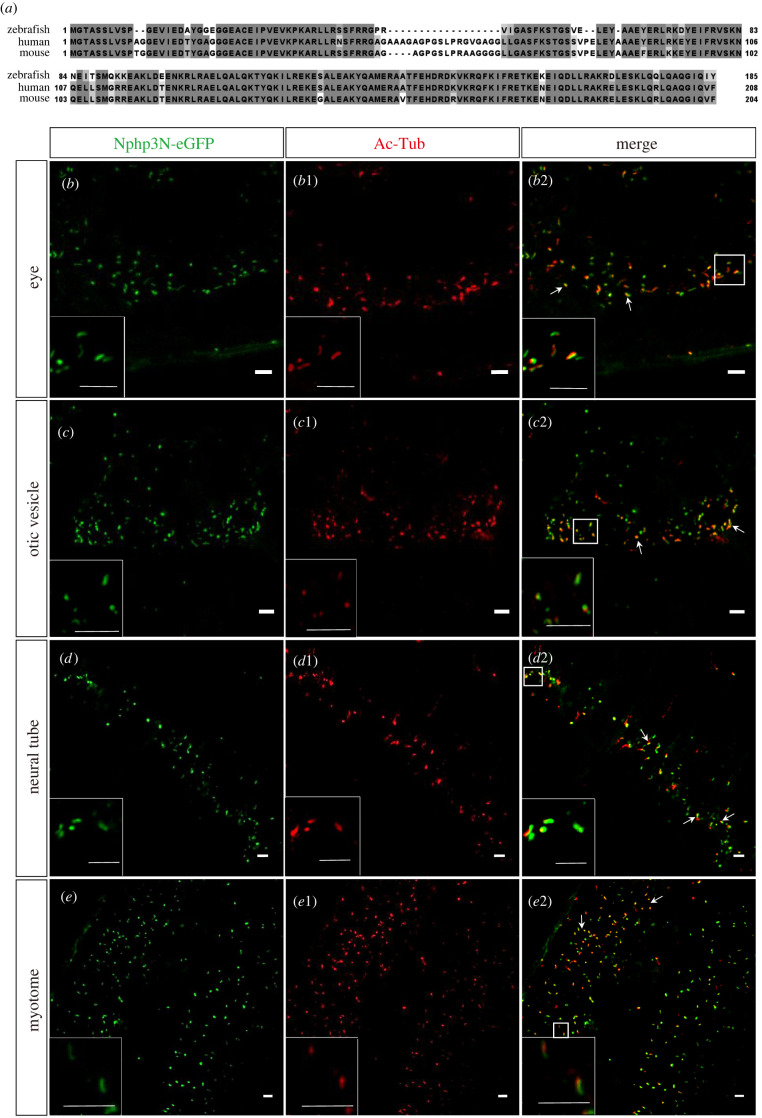
Transient expressed N-terminal peptide of zNphp3 (zNphp3N) fused eGFP localized to PC in zebrafish embryos. (*a*) Sequence alignment of zNphp3N with human and mouse homologs. The Clustal Omega program was used to align protein sequences from *D. rerio* (zebrafish), *H. sapiens* (human) and *M. musculus* (mouse). High and low amino acid similarities are emphasized in dark and light gray, respectively. (*b*–*e*2) At 18 hpf embryos, the transiently expressed fluorescent fusion protein zNphp3N-eGFP was precisely localized in the primary cilia of various cells in the otic vesicle, neural tube, and myotome. (*b–e*) Ciliary localization of zNphp3N-eGFP protein (in green) expressed transiently in zebrafish embryos. (*b*1–*e*1) Cilia labelled by anti-AcTub (red). (*b*2–*e*2) Co-location of transiently expressed zNphp3N-eGFP and cilia labelled by anti-AcTub. Arrows indicate places with obvious co-localization. The frame denotes the enlarged site. Scale bar, 5 µm.

Previous studies showed that a 204 residue N-terminal region of mouse Nphp3, containing a conserved Gly myristoylation site (G2) and CC domain is the minimum peptide required for cilia localization [29]. We found that the first 185 aas of zNphp3 are equivalent to the first 204 amino acids of mouse Nphp3 and the first 208 amino acids in human NPHP3. The zNphp3N shares 86.16% similarity with that of mouse Nphp3N, as well as the critical myristoylation Gly site and the CC domain, suggesting that the zNphp3N may be sufficient for ciliary targeting in zebrafish (figure 2*a*). As predicted, an N-terminal fusion of eGFP to zNphp3N localized to the cilia shaft in various cells of transiently transgenic 18 hpf embryos (figure 2*b–e*). By contrast, mutant zNphp3N (1–185: G2A)-GFP signals were largely restricted to the cytoplasm, with only faint labelling of the basal body (electronic supplementary material, figure S2); this indicates that ciliary shaft targeting is dependent on the N-terminal myristoylation site. Thus, zNphp3N can be used as a PC-specific peptide to drive proteins of interest to the cilia in zebrafish embryos.

### 2.3. Generation of a stable zNphp3N-mCherry transgenic line

**Figure 3.**
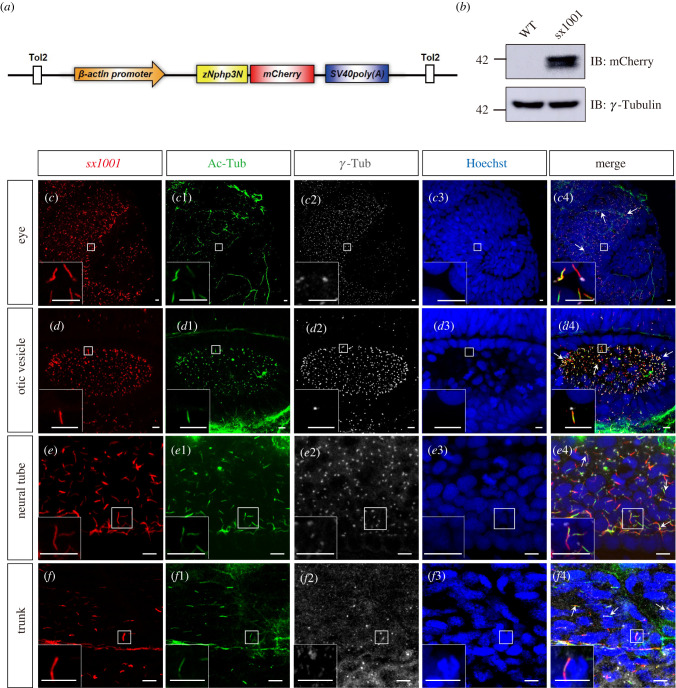
Stable transgenic lines *sx1001* effectively marked the ciliary structure in nearly every embryonic tissue. (*a*) Schematic diagram of transgenic vector construction. (*b*) Western blot image of Nphp3N-protein expression levels. Protein from wild zebrafish embryos was used as the negative control. (*c*–*f*4) The cilia-tagged transgenic zebrafish *sx1001* endogenously produced zNphp3N-mCherry to label the ciliary structure in the eyes, otic vesicle, neural tube and trunk. (*c–f*) Endogenous zNphp3N-mCherry fluorescent fusion proteins (red). (*c*1–*f*1) Cilia labelled by anti-AcTub (green). (*c*2–*f*2) The basal body of cilia marked by anti-γ-tubulin (grey). (*c*3–*f*3) Nucleus stained by Hoechst (blue). (*c*4–*f*4) The merge panel. Arrows indicate places with obvious co-localization. The frame denotes the enlarged site. Scale bar, 5 µm.

To generate a stable line in which the cilium is labeled in all cell types, a *Tol2* transgenic construct carrying *znphp3N-mCherry* driven by the *β-actin* promoter was assembled into *pMiniTol2* plasmid by Gibson Assembly method [30] (figure 3*a*), and introduced into zebrafish embryos by microinjection together with *Tol2* transposase mRNA. Stable integrants were identified by fluorescent microscope screening and six lines were established. The line with the strongest red fluorescence, designated Tg (*β-actin*: *nphp3N-mCherry*)^sx1001^ (*sx1001* hereafter), was used for all subsequent analyses in this study. The *sx1001* fish developed normally and were fertile. The *zNphp3N-mCherry* transgene is maternally expressed as all the embryos from *sx1001* females crossed to wild-type males had strong red fluorescence at 4 hpf. The zNphp3N-mCherry protein expression in *sx1001* was further confirmed by Western blot analysis as shown in figure 3*b*. To analyze the PC labelling further, immunostaining against Ac-Tub was performed in 18 hpf transgenic embryos. As illustrated in figure 3*c–f*, in all cell types analysed, including eye, otic vesicle, neural tube, and myotome, the vast majority of PC labelled by Ac-Tub were also mCherry positive, indicating that the zNphp3N-mCherry is a robust marker of the PC. The zNphp3N-mCherry was evenly distributed along the whole ciliary shaft with only weak cytoplasmic signals outside of PC. We note that a few cilium-like subcellular structures were labelled by mCherry, but were Ac-Tub negative; this reflects an artefact of the Ac-Tub staining.

### 2.4. Integration and expression of *znphp3N-mCherry* in *sx1001* has no discernible effect on PC structure or function

**Figure 4.**
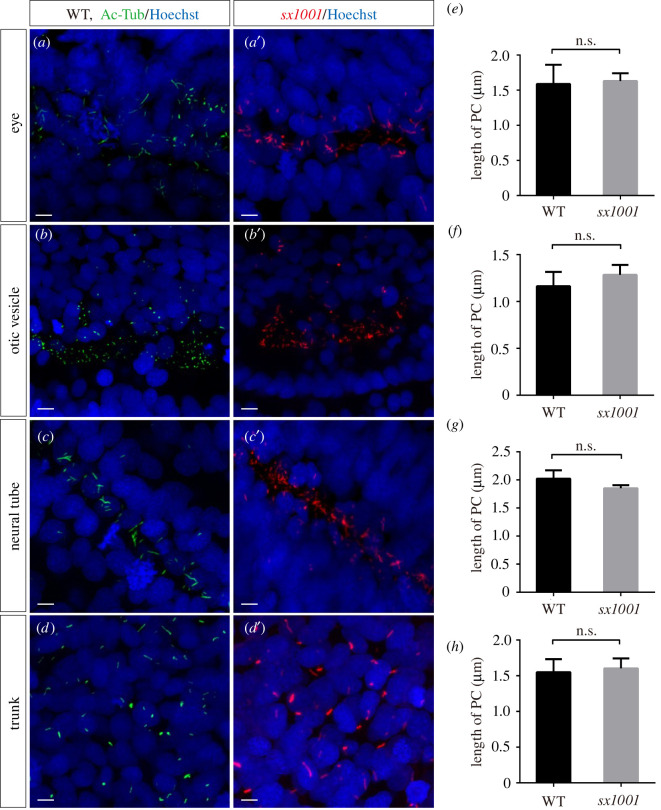
Nphp3N-mCherry integration into *sx1001* has no influence on the cilium morphology. (*a–d*) In all tissues examined, no noticeable difference in cilia morphology was detected between *sx1001* and wild-type embryos. (*a–d*), cilia labelled by anti-AcTub in wild-type embryos at 18 hpf (green). (*a*′–*d*′) Cilia marked by *sx1001* (red); nuclei were labelled by Hoechst (blue). Scale bar, 5 μm. (*e–h*) Comparative measurements of the length of cilia in the eyes, otic vesicle, neural tube and myotome from wild-type (black bar) and *sx1001* (grey bar) using the maximum intensity projection (MIP) method [33]. Each sample has been measured the length of 20 straight cilia (*n* = 4 for each sample). Unpaired Student's *t*-test was used for analysis (n.s., not significant).

Since the abnormal expression or loss of full-length zNphp3 leads to cilia abnormalities [31,32], we investigated whether sustained expression of the zNphp3N-mcherry fusion protein in embryos impacts the growth and development of transgenic animals, and/or cilia morphology and function. As previously mentioned, no abnormal phenotypes were found among *sx1001* transgenic animals at either 24 hpf, 5 dpf or adulthood (*n* > 5). Nor was significant developmental delay observed (electronic supplementary material, figure S3). No obvious morphological defects were observed in the PC of *sx1001* embryos at 18 hpf compared to wild-type in all tissues analysed (figure 4*a–d*). In addition, the length of PC in the eye, otic vesicle, neural tube, and myotome showed no significant difference from wild-type (figure 4*e–h*). These results indicate that neither the integration nor expression of the *znphp3N-mCherry* transgene have deleterious effects, making the transgenic line suitable for investigating structure and function.

As further confirmation of the gratuitous nature of the tagged peptide, we investigated Hh pathway activity in *sx1001* animals. No significant effects on the expression of *ptch1* and *ptch2*, two critical Hh pathway target genes, were detected by qRT-PCR in *sx1001* compared to wild-type (electronic supplementary material, figure S4A-B). Additionally, the Hh-dependent specification of muscle cell types was analysed in *sx1001* embryos. The superficial slow fibres (SSFs), muscle pioneer cells (MPs) and medial fast fibres (MFF) which are specified by different levels of Hh signalling activity [34], all appeared normal, as revealed by Prox1a+, Eng2a+ and double-positive cells at 24 hpf, respectively (electronic supplementary material, figure S4C–J). Taken together, these data support the conclusion that the transgenic integration and ciliary accumulation of Nphp3N-mCherry in *sx1001* has no obvious impact on the structure or functions of PC.

### 2.5. *Sx1001* facilitates live imaging of cilia

**Figure 5.**
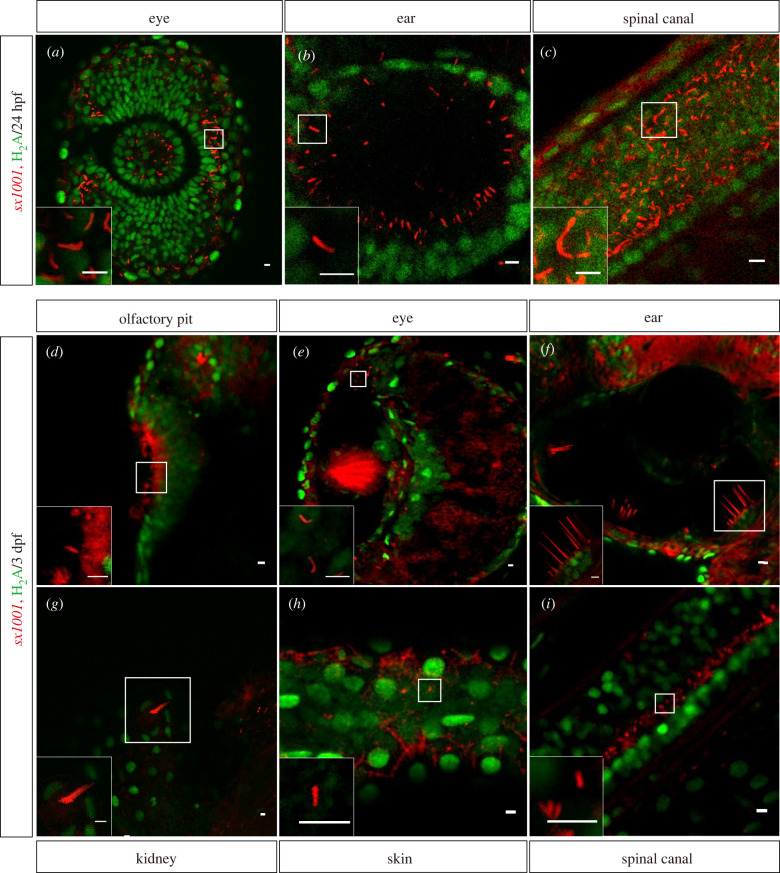
*sx1001* enabled the live imaging of cilia at the tissue level. (*a–c*) In living *sx1001* at 24 hpf, the cilia of the embryo were clearly visible in red fluorescence in the retina, otic vesicle, and spinal canal. Histone2A with C terminal fused eGFP was used to label the nucleoli in live embryos. In live 3 dpf *sx1001* larvae, the various ciliary structures have been revealed and identified with a red fluorescent signal from different tissues as indicated (*d–i*). The frame denotes the enlarged site. Scale bar, 5 µm.

We next asked whether the *sx1001* line is suitable for imaging cilia in live embryos and larvae. Firstly, the PC of *sx1001* embryos at 24 hpf were examined. Consistent with the findings in fixed tissue, the PC in the retina, otic vesicle and neural tube in *sx1001* clearly expressed red fluorescence (figure 5*a–c*). Similarly, in live 3 dpf *sx1001* larvae, the different ciliary structures were also labelled (figure 5*d–i*). For example, zebrafish have both motile and non-motile cilia, and both types of cilia were clearly labelled in *sx1001*. The kinocilia of the inner ear, which are preserved throughout adulthood and play a role in hearing, were also visible in live *sx1001* larvae. In conclusion, *sx1001* can precisely and in real-time display the status of different cilia in a variety of cell types *in vivo*.

### 2.6. Cilia in adult tissues of *sx1001* are uniformly labelled

**Figure 6.**
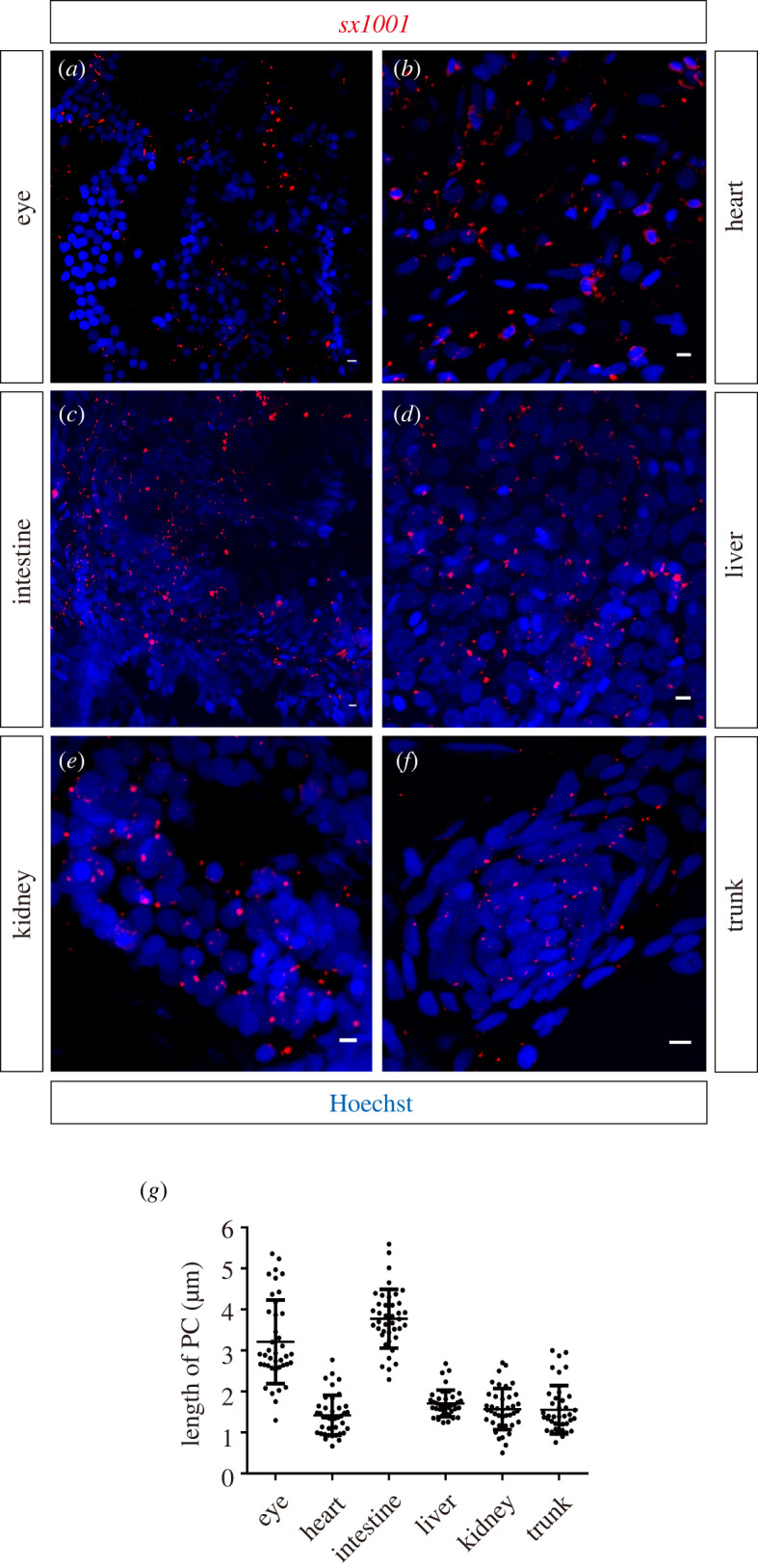
Cilia in adult *sx1001* tissue were uniformly labelled. (*a–f*) Transverse cryosection of different tissues to observe the distribution of *sx1001* labelled (red) cilia in adult zebrafish (*n* = 3 for each sample). The nucleus was stained by Hoechst (blue). Scale bar, 5 μm. (*a*) Cilia at the photoreceptor cell layer; (*b*) cilia of myocardial cell cilia; (*c*) cilia structure of the small intestine; (*d*) cilia of liver cells; (*e*) cilia of renal cell; (*f*) cilia of skeletal muscles. (*g*) The length of tissue cilia was measured using the MIP method previously described. Each sample has been measured with the length of 40 straight primary cilia (*n* = 5 for each sample).

Having shown that *sx1001* efficiently labels various cilia in embryos, we next investigated if this property persists into adulthood. To observe the distribution of labelled cilia in adult fish, we examined various organs including the eye, brain, heart, intestine, liver, kidney and muscle by cryosectioning and immunofluorescence staining. Cilia labelled by zNphp3N-mCherry were observed in all these organs (figure 6*a–f),* varying in number and length. Although cilia could be seen in all types of vertebrate cells, not all cells are ciliated due to their differing cell cycle status. More and longer cilia were observed in the intestine and retina, while fewer and shorter cilia were found in heart, liver, kidney and somatic muscles (figure 6*g*). This variation in cilia length and number may reflect functional differences among these tissues.

### 2.7. *Sx1001* facilitates *in vivo* analysis of vertebrate Hh signalling

**Figure 7.**
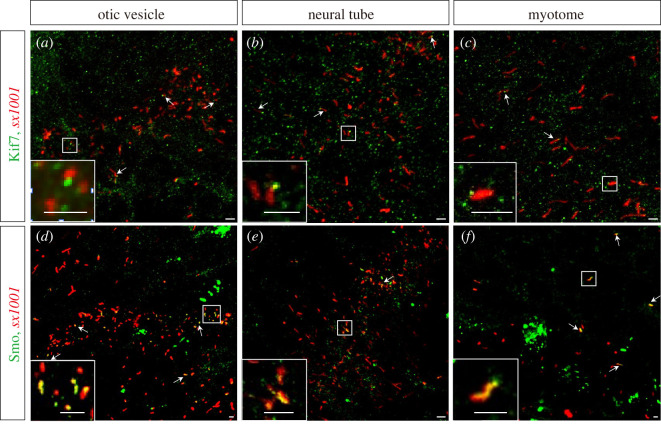
*sx1001* is an effective tool for studying the vertebrate Hh-related components. (*a*–*c*) Kif7-eGFP fusion proteins (green) accumulated at the tip of cilia in the otic vesicle, neural tube, and myotome in *sx1001* (red). (*d*–*f*) Smo-GFP fusion proteins (green) were observed across the ciliary raft in the otic vesicle, neural tube, and myotome in *sx1001* (red). The frame denotes the enlarged site. Scale bars, 2.5 µm.

Most of the key components of the Hh pathway, including PTCH, SMO, KIF7, SUFU and GLI, are regulated through translocation to the PC. The *sx1001* line provides the possibility of monitoring this translocation *in vivo*. Fluorescently tagged Kif7-GFP and Smo-GFP fusion proteins were transiently expressed in *sx1001* embryos. Consistent with previous studies of fixed specimens, both fusion proteins showed specific ciliary localization [35–37]. The Smo-eGFP was distributed throughout the shaft of the PC, while Kif7-eGFP accumulated at the tip of the PC in otic vesicles, neural tube and myotome (figure 7*a–f*). These findings illustrate the utility of the *sx1001* line for real-time monitoring of Hh-pathway components in a living organism.

## 3. Discussion

Nphp3 is a ciliary protein that has been reported to function in the maintenance of cilia number and cAMP levels [32,38]. The zebrafish Nphp3 (*znphp3*) shares 204 aas with mouse Nphp3 and 208 aas with human NPHP3. Although the functions of z*nphp3* in development at embryonic stages have been studied using morpholino-mediated knockdown, its spatio-temporal expression pattern has not previously been reported. Our qRT-PCR and ISH analyses dectected *znphp3* transcript at 4 hpf, suggesting a maternal contribution of zNphp3 protein. Subsequently, the expression of *znphp3* dramatically increased after 24 hpf, implying an important function at these stages. Consistent with this, knockdown of *znphp3* has been shown to result in body curvature, hydrocephalus, positional inversion and pronephric cysts [32]. In line with its function in the kidney, we observed weak expression of *znphp3* in the pronephric glomerulus. Strong expression in the horizontal myoseptum and somite boundaries is consistent with a function in muscle development, potentially in connections between muscle fibres. Expression in muscle is not uniform, as no signal was seen in the first few somites or in the caudal somites.

By contrast to the signal peptides of secreted proteins or the nuclear localization signals of nuclear proteins, ciliary targeting sequences seem quite diverse. Even the ciliary targeting peptide (CTP) in the third intracellular loop of the GPR161 and 5HT_6_ GPCR proteins is not conserved between these two ciliary proteins, despite their belonging to the same family [20,28,39]. Thus, to date, labelling cilia for *in vivo* monitoring has relied upon the use of whole fluorescently tagged cilia proteins, such as ARL13b and 5HT_6_. As these fusion proteins are functional, their overexpression is likely to affect the status of cilia. Therefore, we explored the use of the N terminal peptide of zNphp3 fused with eGFP as an alternative gratuitous marker of cilia. Our finding that Hh pathway activity is unaffected by expression of this fusion protein in *sx1001* transgenic fish, supports the view that it has no deleterious effects on cilia function. In addition, we have tested both zNphp3N-eGFP and zNphp3N-mCherry fusions, and found that both localize to the whole ciliary shaft. This raises the possibility that zNphp3N could be used to drive any peptide of interest to the cilia structure.

Although no conserved motif for ciliary localization has been identified, there were some clues for the conservation of the ciliary localization signal. The first eight amino acids of Nphp3N are known to be substantially conserved among vertebrates. This sequence matches the recognition sequence for N-myristoyltransferase: M-G-XX-X-S/T-. The GFP signals from the myristoylation site mutant, in which the second glycine was replaced with alanine, were lost in the ciliary shaft but persisted in the cytoplasm [29]. Conserved with mouse Nphp3N, the G2 of zNphp3N is critical for its ciliary localization. Consistently, in our study, a G to A mutation caused an impairment in zNphp3N ciliary shaft targeting (electronic supplementary material, figure S2). This G2 is supposed to be a myristoylation modification, a type of lipid acylation modification. Furthermore, there are also some other lipid modifications, such as prenylation and palmitoylation that are supposed to be important for proper ciliary localization. For example, the palmitoylation modification at the C-terminus of 5-HT is critical for its ciliary trafficking. Interestingly, the conjugated modification by cholesterol at Asp95 of mouse Smo has been reported to be essential for its localization to the PC, suggesting that lipid modification is a shared requirement for ciliary localization. Further investigation should be focused on the identification of the lipid modification types and sites, potential enzymes and the mechanism of ciliary accessing control.

A growing number of studies have identified critical cellular signalling pathways that act through PC. To our knowledge, this is the first time that the zNphp3-specific cilia localization sequence has been used to generate a cilia-tagged transgenic zebrafish line for live-cell dynamic imaging of cilia. This stably inheritable transgenic line not only enables accurate, real-time monitoring of cilia state in multiple cell types *in vivo*, but also represents a powerful tool for functional research of receptors and regulatory proteins in cilia at the output of balanced signalling cascades.

## 4. Experimental procedures

### 4.1. Zebrafish husbandry

Adult fish, AB strain, imported from China Zebrafish Resource Center, was maintained at 28°C on a 14 h light/10 h dark cycle in Shanxi University zebrafish facility. Embryos were collected from natural crosses and raised in E3 media (5 mM NaCl, 0.17 mM KCl, 0.33 mM CaCl_2_ · 2H_2_O, 0.33 mM MgSO · 7H_2_O), and staged under the standard protocol [40]. Phenylthiourea (PTU, 0.003%) was added to inhibit pigment formation for embryos older than 24 hpf.

### 4.2. DNA constructs, RNA synthesis, injection and generation of zebrafish transgenic lines

The transient expression constructs of *pCS2+-npnp3-eGFP* and *pCS2+-npnp3N-eGFP*, and the transgenic construct of *pMiniTol2*-*β-actin*-*npnp3N-mCherry* were generated by Gibson assembly. The primers used for cloning were listed in electronic supplementary material, table S2. The transient expression constructs of *pCS2+-kif7-eGFP*, *pCS2+-smo-eGFP* and *pDB600* were as described [35,41].

For capped mRNA synthesis, the transient expression constructs were linearized by NotI or XbaI for *pCS2+* and *pDB600* plasmids, respectively, then purified by Zymogen DNA concentrator. The mRNA was synthesized by either Sp6 or T3 Invitrogen Ambion mMessagemMachine Kit according to the promoter in the constructs, then was purified by LiCl precipitation.

For transient expression of eGFP fusion protein, 100 pg of the mRNA was injected into one-cell stage embryo and the injected embryos were collected at 18 hpf or 24 hpf depending on the experiment design.

For generating the transgenic lines, the *pMiniTol2*-*β-actin*-*npnp3N-mCherry* was co-injected with Tol2 transposase mRNA into one-cell stage embryos, and the T_0_ was screened at 24 hpf. The embryos with strong red fluorescence were grown as potential founders. The T_0_ were out-crossed with wild-type when grown up, and T_1_ with strong red fluorescence was screened out as the stable transgenic lines.

### 4.3. RNA isolation, RT-PCR and quantitative RT-PCR

Total RNA was extracted from zebrafish embryos using Trizol at different stages (Invitrogen). 1 mg of RNA was then used to synthesize the cDNA using SuperScript IV reverse transcriptase (Invitrogen) and oligodT primers (Sangon Biotech). The RT-PCR was performed using the 2× EasyTaq PCR SuperMix (Trans). For quantitative RT-PCR, about 0.2 µl of cDNA was used and the qRT-PCR was performed using GoTaq qPCR Master Mix (Promega) on BioRad qRT-PCR system. Normalization was done against *β-actin* and primers were listed in electronic supplementary material, table S2.

### 4.4. Whole-mount *in situ* hybridization

The C terminal of zebrafish *nphp3* including parts of its 3′UTR was cloned to pGEM-T easy (Promega) as the RNA probe template. The digoxigenin-labelled *nphp3* RNA probe was synthesized by Roche RNA labelling Kit. *In situ* hybridization was carried out as previously described [42]. The images were taken by Imager M2 microscopy.

### 4.5. Western blot analysis

The embryo was lysed in lysis buffer (20 mM Tris HCl, pH 7.4; 150 mM NaCl; 1% TritonX-100; 10% Glycerol; 2 mM EDTA; 1 mM PMSF). Samples were microcentrifuge for 20 min at 4°C, loading buffer (37.5 mM Tris HCl, pH 7.4; 3% SDS; 0.01% bromophenol blue; 6.25% glycerol; 100 mM DTT) was added to the supernatant and the equivalent of 30 embryos run on each lane of a 10% acrylamide denaturing gel, and electroblotted onto Immobilon-P polyvinylidene fluoride (PVDF) membrane (Millipore). Rabbit anti-mCherry (1 : 1000; Sigma) or rabbit anti-γ-tubulin (1 : 3000; Sigma) were diluted in blocking buffer and incubated with membranes at 4°C overnight. Membranes were incubated with HRP conjugated secondary antibody for 1 h at RT. Proteins were detected with an infrared imager (GE Healthcare).

### 4.6. Cryosection and immunofluorescence

Fresh tissues were dissected from 3 months of zebrafish, then rinsed quickly with cold PBS. After 2 h of fixing in 4% paraformaldehyde, tissues were embedded in 2% low-melting agarose and incubated in 30% sucrose at 4°C overnight. The samples were cryosectioned with the slice thickness of 20 µm, and then proceeded to immunofluorescence. Specific immunostaining methods were performed as described [43]. Hoechst was used to stain the nucleus, see electronic supplementary material, table S1 for exact dilution concentrations.

For antibody immunostaining at embryonic stages, embryos were fixed in 4% paraformaldehyde at room temperature for 2 h and then were permeabilized in acetone at −20°C for 7 min, then quenched. After 1 h of incubation in blocking solution (DPBS, 1% BSA, 1% DMSO, 0.5% TritonX-100) at room temperature, primary antibodies diluted in blocking solution were added as described in electronic supplementary material, table S1 and the incubation was performed overnight for incubation at 4°C. After subsequent washes in DPBS-Triton, embryos were incubated with Alexa conjugated secondary antibodies (Alexa Fluor 488 anti-mouse and Alexa Fluor 647 anti-rabbit) and Hoechst overnight at 4°C. Again, Embryos were washed several times in DPBS-Triton and soaked in DPBS with 70% glycerol for imaging. All of the antibodies used in this study, see electronic supplementary material, table S1. Fluorescence was visualized on an LSM 710 confocal microscope with 63× oil immersion objective, 40× and 20× objective. Digital images were acquired using ZEN software.

### 4.7. Statistics

All experiments were replicated at least three times. The experiment results were presented as the mean ± standard deviation. A one-way analysis of variance (ANOVA) and *t*-test was used to calculate *p*-values via GraphPad Prism 7.0 software. The value *p* < 0.05 was considered statistically significant.

## Data Availability

The data are provided in electronic supplementary material [44].
